# Sonographic Assessment of Fetal Anatomy in the First Trimester: A Comparative Study of Visualization Rates Using Two- and Three-Dimensional Ultrasound

**DOI:** 10.3390/clinpract16070132

**Published:** 2026-07-15

**Authors:** Savoia Fabiana, La Verde Marco, Giudicepietro Antonia, Minnella Gian Piero, Fantasia Ilaria, Sarno Laura, Quaresima Paola, Gerbino Martina, Volpe Grazia, Dall’Asta Andrea, Morlando Maddalena

**Affiliations:** 1 Department of Obstetrics and Gynecology, University Luigi Vanvitelli, 80138 Naples, Italy; 2 Hospital Sant’Anna e San Sebastiano, 81100 Caserta, Italy; 3 Department of Obstetrics and Gynecology, University Paolo Giaccone, 90127 Palermo, Italy; 4 Obstetrics & Gynecology Unit, Hospital San Salvatore, 67100 L’Aquila, Italy; 5 Department of Neurosciences, Reproductive Science and Dentistry, University Federico II, 80138 Naples, Italy; 6 Department of Obstetrics and Gynecology, University Magna Graecia, 88100 Catanzaro, Italy; 7 Department of Obstetrics and Gynecology, Hospital Santa Croce e Carle, 12100 Cuneo, Italy; 8 Department of Fetal Medicine and Surgery, University of Milan Cà Granda, 20122 Milan, Italy; 9 Department of Obstetrics and Gynecology, University of Parma, 43126 Parma, Italy

**Keywords:** first trimester anatomy, first trimester scan, 2D ultrasound, 3D ultrasound, early anomaly scan

## Abstract

**Background/Objectives**: This study aimed to compare the visualization rates of fetal anatomical structures with standard 2D ultrasound examination versus a single 3D volume acquired during the first trimester. **Methods:** This multicenter prospective study was performed in nine tertiary referral centers, by experienced sonographers. A standard protocol was adopted in both 2D and 3D modalities to assess the 17 anatomical structures listed in the national and international guidelines. The included cases were non-anomalous fetuses from women booked for combined screening test between 11 + 0 and 13 + 6 weeks. **Results:** Two hundred and thirty-nine women were included in the study. The mean gestational age at ultrasound examination was 12 weeks ± 5 days (±5.4 SD). All the 17 structures were seen in 155/239 fetuses (64.85%) at 2D evaluation and in 84/239 (35.15%) at 3D evaluation (*p* = 0.001). Comparing 2D and 3D visualization, the following anatomical structures showed a statistical difference: posterior fossa (92% vs. 74%, *p* < 0.005); neck (94.2% vs. 87.2%, *p* < 0.001); orbits (93% vs. 85.6%, *p* < 0.001); nasal bone (93% vs. 88.8%, *p* = 0.016); lung fields (99.5% vs 96.7%, *p* = 0.046); cardiac axis (99.5% vs. 83.9%; *p* < 0.001); bladder (97.5% vs. 91.7%, *p* < 0.001); abdominal wall (98.7% vs. 94.2%, *p* < 0.001). For the other anatomical portion, no statistically significant differences were found. **Conclusions:** Our study demonstrates that a full assessment of fetal anatomy is best performed using 2D ultrasound, while 3D ultrasound showed lower overall visualization rates, with comparable performance for selected structures, suggesting a potential supplementary use in specific clinical contexts.

## 1. Introduction

Over the last decades, prenatal diagnosis has shifted from the second to the first trimester owing to advances in ultrasound (US) imaging with enhanced resolution and image processing [1,2], high-resolution transabdominal (TA) and transvaginal (TV) transducers, widespread first trimester risk assessment with improved practitioner’s skills and increased knowledge of developmental fetal anatomy and placental function [3,4]. The gestational window between 11 + 0 and 13 + 6 weeks provides an opportunity for accurate pregnancy dating [5] and fetal aneuploidy risk assessment [6]. In Italy, the combined screening test includes the nuchal translucency (NT) measurement and the blood test for PAPP-A and free b-hCG, which is offered to the general population. The primary aim of the combined test is the risk assessment of fetal aneuploidy. According to Syngelaki et al., at the time of NT measurement, an accurate early anomaly assessment can also be achieved using both TV and TA ultrasound [7]. However, the study of fetal anatomy remains an objective of second trimester scan between 19 and 22 weeks of gestation. Early anatomical assessment offers several advantages, including the early detection of major anomalies, reassurance in high-risk pregnancies (e.g., those with a history of previous structural abnormalities), genetic testing and diagnosis at an earlier gestational age, and opportunities regarding time for making reproductive choices [8,9]. Furthermore, three-dimensional (3D) US technology enables the acquisition of a 3D volume for multiplanar reconstruction, which can be used to reconstruct planes and to visualize structures not identifiable on conventional US examination [10]. Three-dimensional volumes can be stored, displayed off-line and analyzed later [10], providing additional opportunities for education and training. In addition, 3D volume acquisition allows long-term storage for research and audit purposes. For two decades ago, 3D volume ultrasound was described as a faster and equally effective support for 2D imaging during the second trimester ultrasound study [11]. Despite these technological advances, inadequate data exists on 2D and 3D ultrasound during the first-trimester anatomical study. This multicenter prospective study aimed to compare the visualization rates of fetal anatomical structures obtained by routine 2D ultrasound and 3D volume acquisition at 11–13 + 6 weeks of gestation.

## 2. Materials and Methods

A multicenter prospective study was conducted in the following nine tertiary referral centers in Italy: University of Campania Luigi Vanvitelli (Napoli), Hospital Sant’Anna e San Sebastiano (Caserta), University of Palermo Paolo Giaccone (Palermo), Hospital San Salvatore (L’Aquila), University of Naples Federico II (Napoli), University of Catanzaro Magna Graecia (Catanzaro), Hospital Santa Croce e Carle (Cuneo), University of Milan and Hospital Cà Granda (Milano), and University of Parma (Parma). The study was performed from December 2022 to April 2023. Local ethics committee approval was obtained before the centers’ enrolling (Ethical Committee “University L. Vanvitelli” protocol number 58/2018, approval date: 13 February 2018). All participants provided written informed consent. Each center, according to its own resources and case-series, enrolled consecutive eligible cases during the study period. The study had an exploratory nature, and no sample size calculation was conducted. All consecutive eligible women with singleton pregnancies scheduled for the combined screening test between 11 + 0 and 13 + 6 weeks were included in the study without missing data. For all patients, a detailed anatomical examination according to the ISUOG guidelines was performed [5]. Current guidelines recommend two types of early anatomical examination [5]: a basic fetal anatomical study and an advanced examination in selected cases, such as increased NT measurement (>3.5 mm), suspected fetal abnormalities on ultrasound, or after genetic counseling [5]. All ultrasound tests were accomplished by physicians certified by the Fetal Medicine Foundation. All the sonographers were trained in the advanced protocol, which was offered to all the patients, including those at low risk, for study purposes. We excluded multiple pregnancies and pregnancies with fetal structural and chromosomal abnormalities. Gestational age was calculated from the menstrual history and confirmed by measuring fetal crown-rump length (CRL). The first step included CRL, biparietal diameter (BPD), nuchal translucency (NT), internal translucency (IT), fetal heart rate (FHR), ductus venosus and tricuspid valve. After this step, all the women included a detailed anatomical examination following the study protocol. The anatomical examination aimed to assess the following seventeen structures: cranial bones, midline falx and choroid plexuses, posterior fossa with three anechoic spaces, neck, profile, nasal bone, eyes with lenses, spine, heart (cardiac axis and four-chamber view with two symmetrical ventricles), symmetrical lung fields, stomach, bladder, kidneys, umbilical cord insertion, and upper and lower limbs with three segments. A transvaginal scan was performed if transabdominal ultrasound was unsatisfactory due to maternal characteristics (e.g., BMI or prior abdominal surgery) or unfavorable fetal position. After the 2D study, a single 3D volume was acquired in a mid-sagittal view of the fetus in a neutral position. Neutral position was considered the same as that recommended for CRL and NT measurements. Ultrasound examinations were performed using high-end equipment at all centers, equipped with a 3.5–5 MHz transabdominal probe and a 4.5–11.9 MHz transvaginal probe. The volumes acquired were subsequently analyzed offline, at a later timepoint, by the same sonographer who performed the acquisition, using multiplanar rendering to assess the above-mentioned anatomical structures. The same sonographer performed the 3D offline analysis and the real-time 2D examination. This approach reproduces real-world clinical practice and ensures correct volume interpretation on the part of the sonographer.

The statistical analysis was conducted using RStudio Desktop version 2023.12.1+402 (Posit Software, PBC, Boston, MA, USA). Descriptive statistics were used to summarize the baseline characteristics of the fetuses. Continuous variables were presented as mean ± standard deviation (SD), and categorical data were reported as numbers and percentages. A McNemar’s test was used to analyze the paired categorical data. A regression model was fitted to assess the relationship between the dependent variables and the predictors CRL, NT, BPD, IT, and FHR. The model was executed using the glm() function with a binomial family. Logist regression was performed considering that all the outcomes included were binary (yes/no). A *p*-value of ≤0.05 was considered statistically significant for all analyses.

## 3. Results

During the study period, 239 women with singleton pregnancies between 11 + 0 and 13 + 6 weeks of gestation agreed to participate and were included in the study. Table 1 presents the baseline and clinical characteristics of the women included. No significant differences were found in the baseline characteristics across the different centers, including the timing of the examination. The mean gestational age at US assessment was 12 + 5 weeks (±5.4 SD), and the median CRL was 61.4 ± 7.2 mm (Table 1).

The rate of visualization of the 17 anatomical structures comparing 2D and 3D was as shown in full in Table 2. Cranial bones, interhemispheric falx, and choroid-plexus-filled ventricles were always seen in all 239 patients at 2D examination (Table 2). Conversely, at 3D examination, none of the 17 structures were visualized in all the 239 patients. The spine and kidneys were the most challenging structures to evaluate with the lowest visualization rates in both 2D and 3D assessments (Table 2). In more than 90% of cases, at least 65% (11/17) of the structures were successfully visualized by standard 2D examination. When analyzing 3D performance, at least 70% (12/17) of structures were visualized in 60% of cases.

A logistic regression analysis was performed to evaluate the association between the selected variables (CRL, NT, BPD, IT, and FHR) and the likelihood of structure visualization in both 2D and 3D. The results are presented as odds ratios (ORs) with 95% confidence intervals (CIs).

The analysis showed that BPD was associated with an odds ratio (OR) of 1.39 (95% CI: 1.14–1.73, *p* = 0.002), indicating that for each unit increase in BPD, the odds of 2D visibility increase by 39% (Table 3). The analysis showed that CRL was associated with an OR of 1.07 (95% CI: 1.01–1.14, *p* = 0.027), indicating that for each unit increase in CRL, the odds of 3D visibility increase by 7% (Table 4). The other predictors did not have a statistically significant effect on the likelihood of visibility in 2D and 3D.

## 4. Discussion

Our multicenter prospective study demonstrated the superior performance of 2D ultrasound compared with 3D ultrasound in the assessment of first trimester fetal anatomy. Structures such as the posterior fossa, the axis of the fetal heart, and the abdominal wall were more clearly defined with the use of 2D ultrasound. However, for other structures, including the cranial bones, interhemispheric falx, choroid-plexus-filled ventricles, facial profile, spine, stomach, kidneys, and limbs, 3D ultrasound showed similar sensitivity to 2D imaging. In these conditions, 3D images may support the 2D examination, specifically in suboptimal scans [12].

Almost two decades ago, 3D volume ultrasound was already reported as a faster and equally effective alternative to 2D imaging for the evaluation of fetal anatomy in the second trimester [11]. The second trimester remains the gold standard for fetal anatomical evaluation [13]. However, the introduction of NT screening at 11 to 13 + 6 weeks of gestation increased interest in early anatomy assessment [5]. Accurate early anomaly scan requires highly trained and experienced practitioners. In addition, some anatomical structures and abnormalities develop later in gestation (e.g., corpus callosum) and therefore cannot be detected during the first trimester [14]. Some findings may also be inconclusive at an early gestational age, thereby complicating prenatal counseling [5]. A systematic examination including a standardized protocol has been proven to significantly increase the detection rates of anomalies in the first trimester [5]. Current international and national guidelines propose two levels of early anatomical examination at 11 + 0 to 14 + 0 weeks’ gestation [5]: a basic structural survey and a more advanced examination in selected cases, such as increased NT measurement (>3.5 mm), suspected fetal abnormalities on ultrasound, or following genetic counseling [5]. Increased expertise in first trimester screening, combined with a more comprehensive protocol, may allow the earlier detection of a wider spectrum of structural anomalies [15]. Approximately half of the congenital structural defects can now be diagnosed in the first trimester. NT thickening, along with other ultrasound markers, can herald the presence of structural defects, and detailed evaluation of fetal anatomy is becoming widely recognized as a part of the first-trimester ultrasound [3,16]. Early anatomy scans have advantages including: 1. early detection of several major anomalies (for example hydrops, acrania, anencephaly, holoprosencephaly, omphalocele, body stalk anomaly, pentalogy of Cantrell, etc.) [17], 2. early reassurance in high-risk pregnancies, such as those with a history of previous structural abnormalities [18], 3. earlier genetic diagnosis and if necessary termination of pregnancy [19], and 4. additional time for counseling, advanced genetic testing and parental decision-making [3].

Despite the technological developments that occurred in recent decades and the widespread use of 3D ultrasound, we could not replicate in the first trimester the same encouraging results observed in the second trimester [11]. The lower visualization performance of 3D ultrasound observed in our study may be attributed to several factors. First, the 3D technique is a relatively new technique, requiring advanced equipment, and has a learning curve of several months [20]. Technical know-how, spatial reasoning, and continuous training are helpful for those learning the technique [21]. In addition, the 3D volume scan is related to the 2D ultrasound scan [22], meaning that optimal 3D imaging is only achievable when 2D images are good [23,24]. Several cases had incomplete or lower-quality images in 3D compared to 2D images. The quality of 3D volumes and multiplanar analysis can be conditioned by fetal movements; in fact, at this gestational age, fetuses are very active, and more time may be required before an optimal volume is obtained. When analyzing the single-structure visibility, both on 2D and 3D examinations, the lower visualization rates for some structures, such as the spine and kidneys, may reflect the lower confidence of the operator with those structures in the first trimester. Conversely, the visualization of some structures, such as cranial bones, interhemispheric falx and choroid-plexus-filled ventricles, in all the patients in 2D and almost all in 3D may be explained by their routine inclusion in the standard first trimester anatomical protocol. As expected, our study has confirmed that the detection rates for both modalities improved with advancing gestational age, due to larger fetal and organ size. Despite all the limitations, 3D imaging offers advantages compared to the 2D standard study. However, our findings did not support 3D imaging’s superiority, but confirmed it as a complementary support to first trimester anatomic study. It enables a comprehensive review of the acquired volume, without re-scanning the patient directly. Retrospective assessment of stored 3D volumes facilitates a second opinion through a virtual scan [10,20,21,25]. Moreover, digital storing and post-processing provides new opportunities for medical education; in fact, stored 3D volumes can be reviewed by multiple trainees, aiding in fetal anatomy recognition and hands-on learning [22,25,26].

Our study showed several limitations. First, we did not include fetal malformations, with a detection rate for pathological conditions. Second, all the assessments were performed by experienced sonographers, and inter-operator variability was not assessed, which limits generalizability. Third, in our study, we have not evaluated the time required for 2D and 3D ultrasound examinations. Previous studies showed less time to complete a 3D volume assessment at 17–21 weeks [11]. At least, due to the exclusion of fetal abnormality exclusion and perinatal outcomes, our findings should not be considered in terms of diagnostic evaluation. Additional studies are necessary to complete our exploratory analysis.

Despite these limitations, our study has several strengths. We conducted a prospective, multicenter trial, performed by sonographers all certified by the Fetal Medicine Foundation, ensuring a high level of operator competence. Moreover, anatomical assessment has been performed according to a meticulous, standardized protocol, which is associated with greater sensitivity, as demonstrated in a recent review of the literature [14].

Emerging technologies, such as artificial intelligence (AI)-assisted reconstruction and automated volume analysis, may potentially improve the diagnostic performance of 3D ultrasound in the near future [27,28].

## 5. Conclusions

Our study showed that 2D ultrasound for early fetal anatomy study is superior to 3D ultrasound, while 3D ultrasound could support 2D study in specific clinical contexts and for training purposes. Further studies are necessary to evaluate the 3D learning curve in non-tertiary settings, as well as the diagnostic accuracy of 3D imaging for detecting fetal malformations.

## Figures and Tables

**Table 1:** Demographic characteristics of the pregnant women included.

Clinical Characteristics	Mean ± SD
Gestational age, weeks + days	12 + 5 (5.4)
BMI, kg/m^2^	23 (4.23)
CRL, mm	61.4 (7.2)
BPD, mm	20.2 (2.66)
NT, mm	1.51 (0.45)
FHR, bpm	160 (6.87)

Abbreviation: SD, standard deviation; BMI, body mass index; CRL, crown rump length; BPD, Biparietal Diameter; NT, Nuchal translucency; FHR, Fetal Heart Rate.

**Table 2:** Fetal anatomical structures explored in 2D versus 3D.

Fetal Anatomy	2D	3D	*p*-Value
Cranial bones	239 (100)	238 (99.5)	0.47
Interhemispheric falx	239 (100)	236 (98.7)	0.24
Choroid-plexus-filled ventricles	239 (100)	235 (98.3)	0.13
Posterior fossa	220 (92.1)	179 (74.8)	<0.005 *
Neck	225 (94.2)	208 (87.2)	<0.001 *
Bilateral orbits	222 (93.0)	205 (85.6)	<0.001 *
Nasal bone	222 (93.0)	212 (88.8)	0.01 *
Profile	225 (94.2)	224 (93.8)	1.00
Spine	185 (77.3)	186 (77.7)	1.00
Lung fields	238 (99.5)	231 (96.7)	0.04 *
Cardiac axis to left	238 (99.5)	201 (83.9)	<0.001 *
Stomach	238 (99.5)	233 (97.5)	0.07
Bladder	233 (97.5)	219 (91.7)	<0.001 *
Kidneys	164 (68.7)	147 (61.3)	0.05
Abdominal wall	238 (99.5)	225 (94.2)	<0.001 *
Upper limbs	238 (99.5)	238 (99.5)	1.00
Lower limbs	238 (99.5)	233 (97.5)	0.13

Numbers (percentage); * statistically significant (*p* < 0.05).

**Table 3:** Logistic regression analysis on selected variables (CRL, NT, BPD, and FHR) and structure visualization in 2D.

Clinical Feature		95% CI		
	OR	Lower Limit	Upper Limit	*p*-Value
CRL	0.94	0.88	1.00	0.065
NT	1.15	0.60	2.49	0.7
BPD	1.39	1.14	1.73	0.002 *
FHR	1.03	0.98	1.07	0.2

Abbreviations: CRL: crown rump length, BPD: Biparietal Diameter, NT: Nuchal translucency, FHR: Fetal Heart Rate. * *p*-value < 0.05.

**Table 4:** Logistic regression analysis on selected variables (CRL, NT, BPD, and FHR) and structure visualization in 3D.

Clinical Feature		95% CI		
	OR	Lower Limit	Upper Limit	*p*-Value
CRL	1.07	1.01	1.14	0.027 *
NT	0.27	0.09	0.72	0.013 *
BPD	0.90	0.74	1.05	0.2
FHR	1.02	0.97	1.07	0.4

Abbreviations: CRL: crown rump length, BPD: Biparietal Diameter, NT: Nuchal translucency, FHR: Fetal Heart Rate. * *p*-value < 0.05.

## Data Availability

The data presented in this study are available on request from the corresponding author due to privacy or ethical restrictions.

## Abbreviations

The following abbreviations are used in this manuscript:

CRL	crown rump length
FHR	fetal heart rate
BPD	biparietal diameter
NT	nuchal translucency
IT	internal translucency
